# Is There a Role for Patent Medicine Vendors in Tuberculosis Control in Southern Nigeria?

**DOI:** 10.3329/jhpn.v28i6.6605

**Published:** 2010-12

**Authors:** Nkechi G. Onyeneho, Joseph N. Chukwu

**Affiliations:** ^1^ Department of Sociology/Anthropology, University of Nigeria, Nsukka, Enugu State, Nigeria; ^2^ German Leprosy and Tuberculosis Relief Association Enugu, Enugu State, Nigeria

**Keywords:** Patent medicine vendor, Tuberculosis, Nigeria

## Abstract

Patent medicine vendors (PMVs) are a ubiquitous feature of the informal health sector in Nigeria. A previous study on healthcare-seeking behaviour of persons with chronic cough in southern Nigeria found that over 60% of respondents chose the PMV as a healthcare provider of first instance. This study sought to determine the willingness and capability of PMVs to play a role in the national tuberculosis (TB)-control effort. Study sites were selected through a multi-stage sampling process. In total, 388 PMVs, 17 principal officers of PMV associations, and 17 community leaders were purposively selected. Sets of structured questionnaire were administered to the PMVs while information from the principal officers of PMV associations and community leaders was elicited through in-depth interviews and focus-group discussions (FGDs). Quantitative data were collated using the Epi Info software (version 6.04) and analyzed using the SPSS software (version 15). Most (90%) PMVs indicated that they would be ready to cooperate with the national TB-control programme, if trained. Seventy-three percent attended persons with prolonged cough in the course of their career. However, 48% did not know the cause of TB. Only 3% ever-attended a training session on TB control. Sixty-six percent completed at least 12 years of schooling with secondary school certificate. Eighty percent of the community leaders were happy with the work of PMVs. About two-thirds (65.6%) of the PMVs were male. The PMVs are positively disposed to playing roles in TB control. Given this positive disposition and their widespread acceptance in healthcare-delivery in the communities, they have potentials for playing a role in TB control in southern Nigeria.

## INTRODUCTION

There is a growing body of evidence demonstrating the increasing incidence of tuberculosis (TB) in Nigeria with well-developed directly-observed treatment short-course (DOTS) (1–3). Infected persons take a number of options, mainly private-sector operators, before identifying an appropriate DOTS clinic for treatment (4–5).

Persons with undiagnosed TB are reservoirs for high levels of its transmission. Jochem and Walley noted that the contagion parameter suggests that where TB is endemic, each infectious case will result in 20–28 secondary infections (6). Delay in the diagnosis and treatment contributes to worsening complications due to TB.

A recent study on healthcare-seeking behaviours of persons with TB in southern Nigeria revealed that chemists/patent medicine vendors (PMVs) and traditional healers respectively constituted the first port of call for 107 (48.4%) and 27 (12.2%) of the persons with TB in the states studied (2). The number relying on the traditional healers increases as they evaluate the effectiveness of the first options and seek alternative options. They begin to report to the DOTS clinics as the fourth or the last option. This practice is typical of the response pattern to health conditions in most Nigerian societies. For instance, several studies demonstrated that PMVs were the most common source of treatment for malaria in rural and urban communities in Nigeria (7–11).

Despite their prominence in healthcare provision, relatively little is known about PMVs and how they work. Although PMVs are known to belong to PMV associations, there are virtually no published studies on how PMV associations operate. The Pharmacy Law of Nigeria specifies that PMVs should sell only prepackaged patent medicines, which requires that the license be of at least 21 years and submits the names of two referees (12). Within their shops, PMVs have been observed to behave primarily as commercial salesmen, since around 75% simply sell what a customer requests for, and on other rare occasions, fills a prescription (9). In the remainder of the time, the PMV responds to customer requests for advice or a description of symptoms.

Given the massive and complex challenges of case-finding, prompt diagnosis, and providing appropriate and quality treatment for persons with TB in the Nigerian context, it is important to better understand how the informal sector (PMVs) involved in the treatment of TB actually works and identify the pathways of involving them in ensuring prompt diagnosis and appropriate treatment of TB cases.

## MATERIALS AND METHODS

### Study setting

The area is made up of people with very diverse cultural systems, beliefs, and healthcare-seeking practices that are associated with the people's myths about existence and differences in the levels of western education, urbanization, and access to modern health facilities and cultures.

With the promotion of the primary healthcare (PHC) system in Nigeria, communities in each state are linked to a PHC unit that provides services, including disease control and eradication. In these units, there are DOTS services for persons with TB. Along with these formal healthcare-provisioning facilities are a plethora of PMVs and traditional healing homes. The PMVs constitute the primary focus of this study.

### Study area and population

The study focused on the 14 states in the German Leprosy and TB Relief Association-assisted DOTS programme in southern Nigeria. The states are Ekiti, Ondo, and Ogun states in Southwestern zone; Akwa Ibom, Bayelsa, Cross River, Delta, Edo, and Rivers states in South-south zone; and Abia, Anambra, Ebonyi, Enugu, and Imo in Southeast. The 2006 census put that the combined population of these states is over 64,978,686 million. The cross-sectional approach was adopted in collecting quantitative and qualitative data from community members and PMVs in six states in the three geopolitical zones of southern Nigeria.

### Sampling and instruments

The study used multiple instruments and followed a multi-stage random-sampling scheme. One state each was selected to represent each of the major geographic-political zones: Ogun state (Southwest), Akwa Ibom state (South-south), and Enugu state (Southeast). Local Government Areas (LGAs) were stratified into urban and rural LGAs, with one LGA randomly sampled from each stratum, yielding one urban and one rural LGA in each state (6 LGAs in total). In each urban LGA, the LGA headquarter was purposively selected to give typical urban setting for the study, and in each rural LGA, two typical rural communities were randomly selected from a list of all communities in the LGA.

Each study instrument was used as follows:

- In each selected site, the list of all the PMVs in each sampled community was collected from the PMV association. All the PMVs were enlisted in the study and interviewed. In total, 388 PMVs were enlisted and interviewed.
- In each of the LGAs, a PMV association was identified, and 4–7 principal officers were identified (chairman, secretary, treasurer and/or public relations officer) and interviewed on their activities, particularly on their role in the management of TB. In total, 17 PMV leaders were interviewed in-depth in the three states covered.
- In each of the randomly-selected communities, the community leaders were identified and interviewed. In total, 17 community leaders were interviewed in-depth from the selected LGAs.

### Methods of data analysis

Simple descriptive statistics, such as mean, mode, median, and percentage and also graphic illustrations were employed in characterizing data. Correlation analysis was also conducted using non-parametric statistics, such as chi-square (χ^2^), to illustrate the relationship between certain sociodemographic variables and some key issues in the study, such as knowledge of TB and referral practices.

Analysis of qualitative data placed emphasis on the interpretation, description, and recording/writing of what is said. To do this, relevant themes were developed for the coding and sorting of qualitative data, and the Atlas.ti software (version 5.0) was used for managing qualitative data.

## RESULTS

### Sociodemographic characteristics of respondents

More than two-thirds (70.4%) of the PMVs were from the urban areas. Their ages ranged from less than 19 to 45 years and above. About half (49.7%) of the respondents were married (Table 1). Christians (97.4%) dominated the sample of PMVs. About a quarter (25.3%) of the PMVs spent less than five years in the profession. Those who spent 5–9 years constituted 35.3% of the PMVs.

**Table 1.** Distribution of PMVs (n=388) by their sociodemographic characteristics

Sociodemographic characteristics	Rural (n=115)	Urban (n=273)	Total (n=388)
Sex			
Male	84 (73.0)	179 (65.6)	263 (67.8)
Female	31 (27.0)	94 (34.4)	125 (32.2)
Age* (years)			
≤19	-	5 (1.8)	5 (1.3)
20–24	13 (11.3)	32 (11.7)	45 (11.6)
25–29	26 (22.6)	83 (30.4)	109 (28.1)
30–34	29 (25.2)	65 (23.8)	94 (24.2)
35–39	14 (12.2)	32 (11.7)	46 (11.9)
40–44	12 (10.4)	26 (9.5)	38 (9.8)
45+	21 (18.3)	30 (11.0)	51 (13.1)
Marital status			
Never-married	52 (45.2)	35.7	191 (49.2)
Married	60 (52.2)	57.9	193 (49.7)
Married before	3 (2.6)	1.8	4 (1.0)
Religious affiliation			
Catholic	61 (53.0)	120 (44.0)	181 (46.6)
Protestant	25 (21.7)	72 (26.4)	97 (25.0)
Pentecostal	29 (25.2)	71 (26.0)	100 (25.8)
Muslim	-	6 (2.2)	6 (1.5)
Other	-	4 (1.5)	4 (1.0)
Level of education attained			
No formal education	0.(0.0)	9 (3.3)	9 (2.3)
FLSC	11 (9.6)	24 (8.8)	35 (9.0)
WASC/GCE	82 (71.3)	175 (64.1)	257 (66.2)
OND/NCE	19 (16.5)	40 (14.7)	59 (15.2)
HND/first degree	3 (2.6)	21 (7.7)	24 (6.2)
Above first degree	0 (0.0)	4 (1.5)	4 (1.0)
Length (years) in profession			
>5	28 (24.3)	70 (25.6)	98 (25.3)
5–9	40 (34.8)	97 (35.5)	137 (35.3)
10–14	23 (20.0)	66 (24.2)	89 (22.9)
15–19	7 (6.1)	15 (5.5)	22 (5.7)
20–24	14 (12.2)	18 (6.6)	32 (8.2)
25–29	1 (0.9)	3 (1.1)	4 (1.0)
30+	2 (1.7)	4 (1.5	6 (1.5)

Figures in parentheses indicate percentages

*Mean age=33.21 years; mode=28 years; median=32 years; standard deviation±8.832 years;

FLSC=First school-leaving certificate;

HND=Higher national diploma;

NCE=National certificate of education;

WASC=West African school certificate;

GCE=General certificate of education;

OND=Ordinary national diploma

### Knowledge and practice about tuberculosis

More than two-thirds (72.9%) of the PMVs saw people suffering from TB (Table 2). This was confirmed in the qualitative data. A female community leader in Uyo confirmed that TB was common among the people in the communities. She said:

**Table 2.** Distribution of respondents (n=388) by knowledge of tuberculosis

Knowledge of tuberculosis	Rural	Urban	Total
Seen a client with tuberculosis			
Yes	87 (75.7)	196 (71.8)	283 (72.9)
No	28 (24.3)	77 (28.2)	105 (27.1)
Signs and symptoms of tuberculosis			
Cough	44 (38.3)	147 (53.8)	197 (50.8)
Cough with blood	33 (28.7)	73 (27.8)	109 (28.1)
Prolonged coughing (>2 weeks)	32 (27.8)	92 (33.7)	124 (32.0)
Weight loss	17 (14.8)	73 (26.7)	90 (23.2)
Hard cough	15 (13.0)	13 (4.8)	28 (7.2)
Dry cough	19 (16.5)	25 (9.2)	44 (11.3)
Difficult breathing	26 (22.6)	44 (16.1)	70 (18.0)
Change in eye-colour	44 (38.3)	147 (53.8)	191 (49.2)
Weak	33 (28.7)	76 (27.8)	109 (28.1)
Change in skin-colour	32 (27.8)	92 (33.7)	124 (32.0)
Causes of tuberculosis			
Bacterial infection	37 (32.2)	47 (17.2)	84 (21.6)
Witchcraft	5 (95.7)	10 (3.7)	15 (3.9)
Smoking	44 (38.3)	45 (16.5)	89 (22.9)
Dust	29 (25.2)	56 (20.5)	85 (21.9)
Sharing utensils with infected person	22 (19.1)	35 (12.8)	57 (14.7)
Ignorance	15 (13.0)	20 (7.3)	35 (9.0)
Heredity	18 (15.7)	29 (10.6)	47 (12.1)
Poor environment	10 (8.7)	28 (10.3)	38 (9.8)
Poverty	8 (7.0)	31 (11.4)	39 (10.1)
Do not know	28 (24.3)	159 (58.2)	187 (48.2)
Age (years) most at risk			
Children (>5)	2 (1.7)	15 (5.5)	17 (4.4)
Children (5–10)	5 (4.3)	13 (4.8)	18 (4.6)
Children (11–17)	10 (8.7)	8 (2.9)	18 (4.6)
Adults (18–64)	20 (17.4)	19 (7.0)	39 (10.1)
Aged (65+)	16 (13.9)	35 (12.8)	51 (13.1)
All ages equally at risk	33 (28.7)	54 (19.8)	87 (22.4)
Do not know	29 (25.2)	129 (47.3)	159 (40.7)

Figures in parentheses indicate percentages

… I have discovered that there is TB in this community, which disturbs and hinders many people….

Earlier, while enumerating the health problems that confront people in the communities, without prompting, she said:

… I have seen many cases of asthma and also the disease called TB has killed up to five people as I have discovered in this community…. In one household, it killed up to three people.

However, knowledge of TB was not so impressive among the PMVs. Almost half (48.2%) of the PMVs did not know the causes of TB. Ironically, this segment of the respondents was more in the urban area (58.2%) than in the rural area (24.3%). However, more rural PMVs (95.7%) than the urban PMVs (3.7%) subscribed to the existence of supernatural causes of TB. More urban PMVs than their rural counterparts thought that it was a disease of poverty and environment.

Very small proportions (5.2% and 8.5%) of the PMVs sent clients with prolonged cough and suspected TB respectively to any laboratory for diagnosis before treatment (Table 3). Similarly, 57.2% would refer suspected TB cases to higher facilities.

**Table 3.** Distribution of respondents (n=388) by action taken in response to cases

Cases	Treat	Send for laboratory test	Prescribe and treat	Sell drugs as requested	Counsel clients	Refer to higher facility	Dispense drug as prescribed
Fever	227 (58.5)	31 (8.0)	61 (15.7)	29 (7.5)	1 (0.3)	13 (3.4)	26 (6.7)
Malaria	163 (42.0)	57 (14.7)	93 (24.0)	28 (7.2)	6 (1.5)	8 (2.1)	33 (8.5)
Stomach ache	167 (43.0)	36 (9.3)	76 (19.6)	22 (5.7)	3 (0.8)	44 (11.3)	40 (10.3)
Wounds	281 (72.4)	8 (2.1)	17 (4.4)	3 (0.8)	2 (0.5)	48 (12.4)	29 (7.5)
Typhoid	106 (27.3)	120 (30.9)	53 (13.7)	8 (2.1)	3 (0.8)	54 (13.9)	44 (11.3)
Difficult breathing	62 (16.0)	50 (12.9)	31 (8.0)	12 (3.1)	9 (2.3)	188 (48.5)	36 (9.3)
Pneumonia	74 (19.1)	25 (6.4)	76 (19.6)	10 (2.6)	11 (2.8)	143 (36.9)	49 (12.6)
Minor cough	223 (57.5)	7 (1.8)	76 (19.6)	26 (6.7)	-	17 (4.4)	39 (10.1)
Prolonged cough	127 (32.7)	20 (5.2)	188 (48.5)	3 (0.8)	-	31 (8.0)	19 (4.9)
Tuberculosis	58 (14.9)	33 (8.5)	18 (4.6)	-	10 (2.6)	222 (57.2)	47 (12.1)
Asthma	99 (25.5)	21 (5.4)	43 (11.1)	14 (3.6)	9 (2.3)	143 (36.9)	59 (15.2)
Cold and catarrh	232 (59.8)	4 (1.0)	65 (16.8)	21 (5.4)	5 (1.3)	13 (3.4)	48 (12.4)

Figures in parentheses indicate percentages

Most (82.0%) PMVs reported that they treated cases with prolonged cough (Table 4). Table 5 shows that 28.9% of the PMVs treated suspected TB cases in their facilities. This is more in the urban communities (χ^2^=13.898, p<0.001).

**Table 4.** Distribution of respondents (n=388) by treatment of prolonged cough (>2 weeks) and suspected TB cases

Treatment	Rural	Urban	Total
Treatment of prolonged cough			
Yes	98 (85.2)	220 (79.9)	318 (82.0)
No	17 (14.8)	53 (20.1)	70 (18.0)
Treatment of suspected TB cases			
Yes	18 (15.7)	94 (34.4)	112 (28.9)
No	97 (84.3)	179 (65.6)	276 (71.1)
Source of drugs			
Hospital	1 (1.0)	14 (6.5)	15 (4.7)
Open market	95 (96.0)	188 (85.5)	283 (88.7)
Doctors around	67 (67.7)	128 (58.2)	195 (61.1)
Pharmacies	74 (74.7)	133 (60.5)	207 (64.9)
Company representatives	4 (4.0)	24 (10.9)	28 (8.8)
Recognition of fake drugs			
Efficacy on clients	76 (76.8)	144 (66.1)	220 (69.4)
Physical look of drug	16 (16.2)	91 (41.7)	107 (33.8)
NAFDAC number	12 (12.6)	24 (11.1)	36 (11.6)
The source	8 (8.1)	2 (0.9)	10 (3.2)
Usual outcome of treatment for those with prolonged cough			
They all get better	18 (18.4)	50 (22.8)	68 (21.5)
Some get better	63 (64.3)	125 (57.1)	188 (59.3)
Some get worse	6 (6.1)	32 (14.6)	38 (12.0)
All get worse	-	1 (0.5)	1 (0.3)
Cannot say	11 (11.2)	11 (5.0)	22 (6.9)
Usual outcome of treatment for suspected TB cases			
They all get better	2 (11.1)	4 (4.1)	6 (5.2)
Some get better	6 (33.3)	28 (28.9)	34 (29.6)
Some get worse	2 (11.1)	9 (9.3)	11 (9.6)
All get worse	-	3 (3.1)	3 (2.6)
Cannot say	8 (44.4)	53 (54.6)	61 (53.0)
Next step if condition fails to improve after treatment of suspected TB cases			
Change treatment	3 (16.7)	6 (6.1)	9 (7.8)
Increase dosage	-	6 (6.1)	6 (5.2)
Refer clients to other facilities	11 (61.1)	78 (79.6)	89 (76.7)
Do nothing	4 (22.2)	14 (14.3)	18 (15.5)

Figures in parentheses indicate percentages;

NAFDAC=National Agency for Food and Drug Administration and Control;

TB=Tuberculosis

**Table 5.** Distribution of respondents (n=388) by referral practice

Referral practice	Rural	Urban	Total
Likely place to refer a client with prolonged cough (>2 weeks)			
Private hospital	82 (71.3)	222 (81.3)	304 (78.4)
Medical laboratory	32 (27.8)	49 (17.9)	81 (20.9)
Government hospital	15 (13.0)	32 (11.7)	47 (12.1)
Likely place to refer a suspected TB client			
Nowhere	72 (62.6)	246 (90.1)	318 (82.0)
Private hospital	14 (12.2)	24 (8.8)	38 (9.8)
Medical laboratory	0 (0.0)	3 (1.1)	3 (0.8)
Government hospital	30 (26.1)	4 (1.5)	34 (8.8)
Ever-referred client with prolonged cough (>2 weeks)			
Yes	80 (69.6)	221 (81.0)	301 (77.6)
No	35 (30.4)	52 (19.0)	87 (22.4)
Ever-referred a suspected TB client			
Yes	74 (64.3)	182 (66.7)	256 (66.0)
No	41 (35.7)	91 (33.3)	132 (34.0)
Average time between first contact with clients with cough and referral			
<1 week	9 (11.3)	26 (11.8)	35 (11.6)
1-<2 weeks	38 (47.5)	41 (18.6)	79 (26.2)
2–3 weeks	9 (11.3)	64 (29.0)	73 (24.3)
<1 month	7 (8.8)	14 (6.3)	21 (7.0)
>1 month	17 (21.3)	76 (34.4)	93 (30.9)
Reasons why referral may not be made			
If symptom disappears	105 (91.3)	244 (89.4)	349 (89.9)
Client may return to me for treatment	88 (76.5)	204 (74.7)	292 (75.3)
Client may loose confidence in me	88 (76.5)	204 (74.7)	292 (75.3)
Client may not go to referral place	23 (20.0)	46 (16.8)	69 (17.8)
Client may go to other PMVs instead	24 (20.9)	39 (14.3)	63 (16.2)
Client may go to traditional healers instead	25 (21.7)	31 (11.4)	56 (14.4)
I can handle all cases	115 (100.0)	249 (91.2)	364 (93.8)
There are no better facilities around	15 (13.0)	57 (20.9)	72 (18.6)
The people are very poor	35 (30.4)	75 (27.5)	110 (28.4)

Figures in parentheses indicate percentages;

PMVs=Patent medicine vendors;

TB=Tuberculosis

Over half (59.3%) of the PMVs indicated that some clients with prolonged cough they treated got better. Similarly, 5.2% of the PMVs indicated that all the suspected TB cases they treated got better. The large majority (76.7%) of the PMVs referred the suspected TB cases, if they failed to improve.

### Referral practices among patent medicine vendors

Table 5 shows that 78.4% of the PMVs would likely refer clients with prolonged cough (>2 weeks) to private hospitals. Others would refer this category of clients to medical laboratories (20.9%). Those in the urban communities (81.3%) would more likely refer their clients to private hospitals (χ^2^=4.784, p=0.021).

According to the leaders of PMV associations, the PMVs often referred clients to the nearest hospitals when they ever-referred. Incidentally, these nearest hospitals are often the private-for-profit hospitals. In his words:

… If the problem becomes complicated, we tell the person to take him/her to the nearby hospital. These are mostly the private clinics where you may find a doctor….

On actual referral practices, 77.6% of the PMVs had ever-referred their clients with prolonged cough. On the other hand, 66.0% had ever-referred the suspected TB cases. At least one-third of the PMVs waited for more than one month to refer clients with prolonged cough. The average waiting period was 39 days, and this was more among the urban PMVs (χ^2^=30.136; p<0.001).

One of the leaders of the PMV association argued:

… It is the sick, the person with boil who knows where it hurts …. It is the asthma or TB patient who will tell what he has since we are not doctors to administer test and know if it is ordinary cough. So, they are the ones to ask for referral before we refer them….

Table 5 also shows that 89.9% of the PMVs would not refer their clients if the symptoms disappear. Other reasons for not referring clients included the fact that the PMVs feared that the client might not return to their facilities for treatment after the testing (75.3%). All the rural PMVs and 91.2% of their urban counterparts indicated that they would not refer their clients with prolonged cough or those suspected with TB because they can handle all cases.

The figure further reviewed the referral practices of the PMVs on different respiratory health problems. It showed that the referral practices differed with the nature of the respiratory problem. For instance, in the cases of prolonged cough, pneumonia, and chesty cough, large proportions of the PMVs rarely made any referral. In the case of cold and catarrh, a majority (62.5%) of the PMVs never referred their clients. However, in situations where they suspected TB cases, they always referred the patients.

**Fig.**
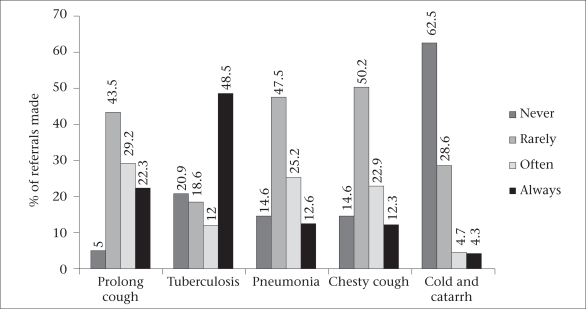
Referral paractices by disease conditions

### Awareness of directly-observed treatment short-course clinics

More than half (58.2%) of the PMVs had never heard of DOTS clinics. Seven in every 10 (65.4%) of those who indicated awareness of DOTS clinics also indicated that they were aware of the existence of DOTS clinic in their LGA. The lack of awareness was more common among the urban PMVs than among the rural PMVs.

Awareness of DOTS clinics also varied among the leaders of PMV associations. For instance, while the Chairman in the Akwa Ibom state indicated awareness, a Secretary from a rural community in the same state indicated ignorance of the existence of the DOTS clinic. The Chairman said:

I have heard about DOTS clinic where people are given drugs to take before the health personnel.… In fact, the first time when I heard about it, I thanked the Federal Government and the organizers of that section immensely….

On the other hand, a Secretary of a PMV association indicated the lack of awareness of the DOTS clinics by saying:

… I have not heard about it (DOTS clinic). I am not aware of DOTS clinic but if it is evolved, it will help in the control of people with tuberculosis in our environment.

Such variations in awareness of the existence and activities of the DOTS clinics were reported in the three states under study. While the urban PMV leaders were aware, their counterparts in the rural communities lacked knowledge of DOTS clinics. Half (50%) of the PMVs indicated that a few people visited the DOTS clinics while another 2.8% of the PMVs indicated that people did not use the DOTS clinics.

The low usage of the DOTS clinics was linked to ignorance (72.2%) and attitudes of the health workers at the DOTS clinics (44.4%). Other reasons were that people were unaware of the existence of DOTS clinics (70.8%) and perceived high cost of testing and treatment at these DOTS clinics (9.7%). These were particularly striking in the rural communities.

On the other hand, many people patronized the PMVs because they are readily available to treat the people (80.7%) and have cheap treatments and drugs (28.4%). They are also friendly (71.1%) and do not delay treatment by first insisting on laboratory testing (22.9%).

According to a PMV, some reasons for being on the ground include, in his words:

… We are in the local communities, a very interior part of the community, and we deal with people of the local communities; hence, such people come to the patent medicine shop to request, “sir, do you have something like this”….

The Public Relations Officer of one of the PMV assocations stated:

Some of them (clients) do not know that they should go for test so that adequate treatment could be known; so, they will come to us thinking we can handle….

### Involvement of patent medicine dealers in detection of TB cases

Table 6 shows that 81.7% of the PMVs agreed that involving the PMVs would increase case-detection. Most (87.1%) agreed that the PMVs would be glad to demonstrate their relevance in disease control. Further, 78.4% agreed that the PMVs would see DOTS clinics as opportunities for free-testing and treatment of their clients. These signify positive disposition towards the involvement of PMVs in TB control.

**Table 6.** Attitudes of respondents (n=388) towards involving PMVs in detection of TB cases by locality

Attitude	Agree	Disagree
Involving PMDs in TB control will increase detection of TB cases in the communities	317 (81.7)	71 (18.3)
Involving PMDs in TB control will make no difference on detection of TB cases	35 (9.0)	353 (91.0)
PMDs will be reluctant to get involved because it will kill their business	42 (10.8)	346 (89.2)
PMDs will be glad to demonstrate their relevance in disease control	338 (87.1)	50 12.9)
PMDs always feel that they can handle TB cases	69 (17.8)	319 (82.2)
PMDs will see DOTS clinic as helping to provide free testing	304 (78.4)	84 (21.6)
Referring clients to DOTS clinics will be seen as delay in action	52 (13.4)	336 (86.6)
PMDs will insist on being paid for referring their clients to DOTS clinics	36 (9.3)	352 0.7)
PMDs are not bothered about testing	31 (8.0)	357 (92.0)
PMDs suspect that referring their clients to DOTS clinics is a way of losing their clients to their rivals, government health workers who actually make money in DOTS clinics	39 (10.1)	349 (89.9)
PMDs will suspect that they might get infected while attending TB clients; therefore, not get involved	57 (14.7)	331 (85.3)

Figures in parentheses indicate percentages;

DOTs=Directly-observed treatment short-course;

PMVs=Patent Medicine vendors;

TB=Tuberculosis

However, on other issues that indicate negative disposition towards the involvement of PMVs in TB control, the PMVs seemed to disagree. For instance, 89.2% of the PMVs disagreed with the idea that the PMVs would be reluctant to get involved in TB control because it would kill their business. Similarly, 90.7% of the PMVs disagreed that the PMVs would insist on being paid for their roles in TB control.

The majority (54.1%) of the PMVs thought that one best way of involving the PMVs in TB control was to train them on detection of TB cases. About one-third (37.4%) indicated that efforts should be made to increase awareness of detection of TB cases among the PMVs. Some PMVs (16.2%) indicated that the PMVs should be supplied with drugs for managing TB.

Many (25.5%) PMVs thought that the PMVs could be engaged in counselling suspected TB cases to go for testing. The majority (60.3%) of the PMVs thought that the PMVs could refer suspected TB cases to the DOTS clinic. Small proportions (13.9%, 4.1%, and 5.9%) opined that the PMVs could treat suspected TB cases, test suspected TB cases in their facilities, and do anything to help control TB respectively.

A very small proportion (2.8%) of the PMVs indicated that they had ever-attended training on TB control. Most (97.2%) had never attended any training on TB control. This situation did not differ between the rural and the urban communities.

The leaders of PMV associations also identified training as one way of getting the PMVs involved in TB control in the communities. One of the leaders in Akwa Ibom state said:

… They (PMVs) should be informed, involved, educated, and trained…. You will know what you have learnt, and if anything comes up, you will tell the person….

The PMVs identified possible barriers to their involvement in TB-control programmes. One of such barriers is the level of training required for the handling of TB on a large scale (77.3%). The most commonly-mentioned barrier, however, was the worry about government regulations (82.0%). The PMVs were also concerned with the attitudes of the health workers in the government health facilities (78.1%).

The PMVs identified some opportunities that could facilitate their involvement in TB control. These included the existing skills they have in handling clients with different ailments, including prolonged cough (100%). The other opportunities mentioned were the existing positive disposition of the community members to the PMVs (88.7%) and the fact that the handling of suspected TB cases is akin to handling other health problems in the communities (77.3%).

The leaders of PMV associations also identified opportunities for mainstreaming the PMVs into an organized TB-control efforts in the communities. One of such opportunities mentioned was the monthly meetings of members of the associations. According to one leader of a PMV association:

… The monthly meetings we have are of great help…. We discuss about various health problems people bring…. I lecture my members in the meeting….

This is typical of the views of the leaders from the other states. Another leader of the PMVs emphasized:

… All these things we are telling in our lectures in the seminars which we always hold. So, we have orientation, this is why you can see me being able to say all these….

### Attitudes of community leaders and leaders of PMV associations towards involvement of PMVs in tuberculosis control

An attitudinal scale was developed to gauge the attitudes of the community leaders and the leaders of PMV associations towards the involvement of PMVs in TB control in the communities. The results revealed that the leaders of PMV associations are generally positively disposed to involving the PMVs in TB control in the communities, irrespective of the state. The Chairman of the PMV association in Ogun state, for instance, agreed that involving the PMVs will help the health system in the communities. He said:

… I agree totally because it will help the rural people. They would not have problems so frequently as now, when they go to the test clinic. … It will help the people greatly…. I agree.

Another leader of a PMV association emphasized:

… Involving the PMVs in the control of TB will make a difference in case-detection because they will effectively care for this person since it is in their neighbourhood.

## DISCUSSION

Respiratory diseases generally and, indeed, prolonged cough which is a sign of TB infection constitute some common health problems taken to the PMVs in the study communities. The study, thus, reviewed the potential roles the PMVs could play in TB control in southern Nigeria. They are highly trainable individuals aged 19–45 years. Awareness and patronage of the DOTS clinic being poor, clients instead visit the PMVs. And the PMVs seldom refer their clients, including those with prolonged cough and suspected TB cases, to private hospitals and other higher facilities but not the DOTS clinics. Referrals are made when the efforts of the PMV fail to ameliorate the condition of the client or when symptoms get worse or when the client demands to be referred. The average waiting period was 39 days.

The implications of such delays are not farfetched. The clients often engage in a process of multiple and varied healthcare-seeking that Auer *et al*. calls ‘shopping’ for diagnosis and treatment therapy (3), typical of poor patients, which often delays diagnosis and the start of treatment, which increases the likelihood of developing multidrug-resistant TB (2, 13).

The PMVs opined that they be trained while the authorities should handle drugs for the management of TB. They identified their existing skills, their acceptance among the community members, and, of course, the similarity of the procedures of handling TB cases and other respiratory problems as opportunities for mainstreaming TB control in the activities of the PMVs.

Earlier studies on healthcare-seeking behaviour of patients revealed that health workers exploit patients and are often unfriendly, especially with poor patients (2, 13–16). Some of them even sell the supposedly free drug. According to some discussants in the FGD sessions, the much-taunted free drugs in health facilities could be likened to the process of obtaining bail for suspects in Nigerian Police cells where bails are officially free but practically paid for (17).

This is in line with other studies. For instance, Uplekar and Shepard and Uplekar and Rangan revealed that people generally associated health workers in public-sector facilities with poor attitude (18–19). Auer *et al*. revealed that, in the Philippines, most TB patients buy the prescribed anti-TB drugs when they visit the DOTS clinics (3). Nichter noted that buying anti-TB medication, rather than receiving it free at the health centre, not only aggravates the financial hardship of the poor, it often results in incomplete and/or irregular intake of drugs (20). These highlight the attitudes of health workers towards patients, which needs improvement if the much-canvassed private-public partnership in healthcare is to be realized (21–22).

### Conclusions

The PMVs have potentials for playing a role in facilitating the detection of TB cases because they are not only trainable, they are skilled in handling other health problems of people in their catchment communities. The PMVs are readily acceptable among the community members. The current referral practices among the PMVs are poor because of the low level of awareness of DOTS clinics in the communities and the PMVs' fear of losing their clients. Another factor is the confidence of PMVs in their ability to handle all cases. The PMVs believe in their ability to handle various health problems of the community members.

## ACKNOWLEDGEMENTS

The German Leprosy Relief Association (GLRA) provided the funds and logistic support for the study. The authors owe the success of this exercise to Gerhard Oehler, GLRA Country Administrator in Nigeria, for making available the necessary financial and logistic requirements for the assignment.
